# Temporal Mapping of CSVD‐Related White Matter Lesions and Concurrent Neurovascular Dysfunction in Spontaneously Hypertensive Rats

**DOI:** 10.1002/cns.71142

**Published:** 2026-09-11

**Authors:** Jing Peng, Jingnian Ni, Fuyao Li, Ting Li, Mingqing Wei, Tianci Ge, Lulu Yao, Jing Shi, Jinzhou Tian

**Affiliations:** ^1^ Department of Neurology, Dongzhimen Hospital Beijing University of Chinese Medicine Beijing China; ^2^ Institute for Brain and Cognitive Disorders Beijing University of Chinese Medicine Beijing China; ^3^ Institute of Traditional Chinese Medicine/National Key Laboratory Beijing University of Chinese Medicine Beijing China

**Keywords:** blood–brain barrier, cerebral small vessel disease, Pde10a/cAMP, spontaneously hypertensive rats, white matter lesions

## Abstract

**Objective:**

To define the critical onset window and mechanisms of white matter lesions (WMLs) in cerebral small vessel disease based on spontaneously hypertensive rat (SHR).

**Methods:**

SHRs and age‐matched Wistar‐Kyoto (WKY) rats were assessed from 20 to 36 weeks of age. Systolic blood pressure, beam‐walking performance, and magnetic resonance imaging (MRI)‐based white matter changes were evaluated at 4‐week intervals. At each time point, randomly selected animals were euthanized for histological analysis. Gait performance and novel object recognition were further assessed at 36 weeks of age. Additional histopathological, neurovascular, neuroinflammatory, transcriptomic, proteomic, and biochemical analyses were performed at the identified critical time point.

**Results:**

SHRs developed sustained hypertension and early small‐vessel morphological alterations from 20 weeks, followed by corpus callosum demyelination at 28 weeks and motor, gait, and non‐spatial memory deficits by 36 weeks. MRI identified 28 weeks as the onset of detectable white matter microstructural impairment, marked by reduced fractional anisotropy, axial diffusivity, and increased radial diffusivity. At this stage, histological and molecular analyses confirmed myelin disruption, altered mature oligodendrocyte (OL)‐ and oligodendrocyte precursor cell (OPC)‐associated marker profiles, microvascular rarefaction, blood–brain barrier disruption, and inflammation in SHRs. Transcriptomic analysis highlighted cAMP pathway dysregulation, whereas proteomic analysis identified Pde10a as a significantly upregulated candidate protein. Suppression of downstream cAMP/PKA/CREB signaling was further supported by biochemical validation.

**Conclusions:**

WMLs emerge at 28 weeks in SHRs and are accompanied by convergent neurovascular injury and inflammatory activation. Pde10a‐associated cAMP signaling may represent a candidate mechanism associated with hypertension‐related WML development in CSVD.

## Graphical abstract

Hypertension induces early neurovascular dysfunction of CSVD, with white matter lesion onset at 28 weeks of age and progression to behavior deficits at 36 weeks of age in spontaneously hypertensive rats. Pde10a/cAMP signaling dysregulation may be related to this process and represent a potential therapeutic target.

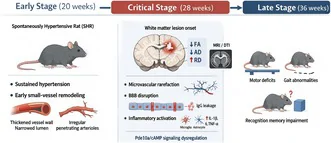

## 1 Introduction

Cerebral small vessel disease (CSVD) is a major contributor to stroke, vascular cognitive impairment, and gait disturbances, with arteriosclerotic CSVD as the most prevalent subtype [1, 2, 3]. White matter lesions (WMLs) are among the most common radiological manifestations of CSVD‐related parenchymal injury [4, 5]. Pathologically, WMLs manifest as demyelination, oligodendrocyte loss, and axonal damage [6, 7]. These parenchymal alterations underpin the core clinical symptoms of CSVD, including executive dysfunction, slowed information processing, and gait disturbances [8, 9, 10]. These symptoms not only significantly impair quality of life but also markedly increase the risk of subsequent serious cerebrovascular events.

Among the various risk factors for CSVD, hypertension stands out as the most prevalent and modifiable factor [11, 12]. Long‐term hypertension damages small vascular endothelial cells and facilitates the extravasation of plasma proteins into the vessel wall, leading to hyalinosis and wall fibrosis. Vascular wall thickening and luminal narrowing reduce cerebral blood flow, ultimately resulting in chronic cerebral ischemia and WMLs [13, 14, 15]. The spontaneously hypertensive rat (SHR) has been adopted as an animal model for CSVD research due to its recapitulation of key disease features, including WMLs, brain atrophy, and cognitive impairment during disease progression [16, 17, 18]. In addition, SHRs may also exhibit broader cardiovascular comorbidity‐related phenotypes that are relevant to CSVD [19]. However, previous studies utilizing the SHR model mainly focused on selected age points or intervention effects; the temporal onset and progression of WMLs and its associated neurovascular and molecular alterations remain insufficiently defined. This knowledge gap impedes a deeper understanding of CSVD pathogenesis and hinders the development of targeted interventions.

In this study, we conducted a serial time‐course study to characterize the progression of WMLs in SHRs from the establishment of hypertension to the later disease stages. Our primary objective was to determine the critical time point for WMLs development using magnetic resonance imaging (MRI) combined with correlative histopathological analysis. We further assessed concurrent neurovascular alterations at this critical stage, including endothelial injury, blood–brain barrier (BBB) disruption, and inflammation. Finally, integrated transcriptomic and proteomic analyses were employed to identify candidate molecular mechanisms associated with WML onset, followed by targeted validation of the key pathway emerging from multi‐omics screening. Together, this study aimed to provide new insights into the temporal evolution and molecular basis of WMLs in SHRs and to identify potential time windows and molecular candidates for future therapeutic intervention.

## 2 Materials and Methods

### 2.1 Animals and Study Design

All animal procedures were approved by the Experimental Animal Center of Beijing University of Chinese Medicine (Approval No. BUCM‐2024053104‐2226) and complied with the ARRIVE guidelines. Forty male SHRs and 4 age‐matched male Wistar‐Kyoto (WKY) rats were used. Animals were housed under standard conditions with free access to food and water.

A serial time‐course study was performed to define the critical time point for WMLs development in SHRs from 20 to 36 weeks of age. At each scheduled time point (20, 24, 28, 32, and 36 weeks), animals were selected from the available pool using a random‐number table before outcome assessment. Non‐terminal measurements were performed in the selected animals, whereas animals assigned to terminal histological or molecular analyses were euthanized and were not used at subsequent time points. At each time point, systolic blood pressure, beam‐walking performance, and brain MRI were assessed. Moreover, a subset of animals was collected for histological assessment. At 36 weeks, gait and recognition memory were further assessed using the CatWalk XT system and novel object recognition test, respectively. After the critical time point for WMLs onset was identified, ultrasound localization microscopy imaging was performed, and the brain tissues were collected for histopathological, neurovascular, inflammatory, biochemical, transcriptomic, and proteomic analyses. Detailed animals number are provided in the Methods S1.

### 2.2 Physiological and Behavioral Assessments

Systolic blood pressure was measured in conscious rats using a noninvasive tail‐cuff system. Motor coordination was assessed using the beam‐walking test [20], and gait parameters were analyzed with the CatWalk XT system (Noldus, Netherlands). Non‐spatial recognition memory was evaluated using the novel object recognition test, and the discrimination index was calculated as follows: the time spent exploring the novel object divided by the total exploration time.

### 2.3 MRI Acquisition and Analysis

Longitudinal MRI was performed on a 9.4‐T animal scanner under isoflurane anesthesia. T2‐weighted imaging was used for brain volumetric assessment, and diffusion tensor imaging (DTI) was used to evaluate white matter integrity. Fractional anisotropy (FA), mean diffusivity (MD), axial diffusivity (AD), and radial diffusivity (RD) were quantified in the corpus callosum [21, 22, 23, 24]. Total brain volume was reconstructed from T2‐weighted images using 3D Slicer software. Detailed acquisition parameters are provided in the Methods S1.

### 2.4 Histological, Immunostaining, and Ultrastructural Analyses

To assess white matter pathology and vascular alterations, brain sections were subjected to Luxol fast blue (LFB) staining and hematoxylin–eosin (HE) staining. Immunofluorescence was used to detect myelin basic protein (MBP), glutathione S‐transferase pi (GST‐pi), neuron‐glial antigen 2 (NG2), cluster of differentiation 31 (CD31) with endogenous IgG, and phosphorylated cAMP response element‐binding protein (p‐CREB), whereas immunohistochemistry was performed for ionized calcium‐binding adaptor molecule 1 (Iba1), glial fibrillary acidic protein (GFAP), and phosphorylated protein kinase A (p‐PKA). Images were acquired under identical settings and quantified using Fiji ImageJ v.1.52p (NIH, USA). For ultrastructural evaluation of myelin and axons, transmission electron microscopy was performed on corpus callosum samples. Detailed procedures and antibodies are provided in the Methods S1.

### 2.5 Western Blot and ELISA

Protein extracts from the corpus callosum were analyzed by western blot for MBP, phosphodiesterase 10a (Pde10a), p‐PKA, cAMP response element‐binding protein (CREB), and p‐CREB, with GAPDH or β‐actin used as internal controls. ELISA was used to measure serum interleukin‐1 beta (IL‐1β), interleukin‐6 (IL‐6), tumor necrosis factor‐alpha (TNF‐α), as well as cyclic adenosine monophosphate (cAMP) levels in the corpus callosum, according to the manufacturers' instructions. Detailed procedures and antibody information are provided in the Methods S1.

### 2.6 Ultrasound Localization Microscopy

Ultrasound localization microscopy (ULM) was used to generate super‐resolved maps of the cerebral microvasculature. Rats were anesthetized, and a cranial window was prepared for imaging. After intravenous administration of microbubble contrast agent, raw ultrasound data were acquired and reconstructed into vascular maps using the manufacturer's software. Vascular density was quantified with Fiji ImageJ as previously described [25]. Detailed imaging settings are described in the Methods S1.

### 2.7 Transcriptomic, Proteomic, and Integrative Analyses

For transcriptomic analysis, total RNA from the corpus callosum was extracted, sequenced, and analyzed to identify differentially expressed genes (DEGs). For proteomic analysis, total protein was extracted from corpus callosum tissue and subjected to data‐independent acquisition proteomics to identify differentially expressed proteins (DEPs). To integrate the two omics datasets, molecules with concordant expression trends were retained for functional enrichment analysis, and key candidates were subsequently validated by RT‐qPCR and western blot. Detailed library preparation, sequencing, database searching, and filtering parameters are provided in the Methods S1.

### 2.8 RT‐qPCR

Total RNA from corpus callosum tissue was reverse‐transcribed into cDNA, and quantitative PCR was performed using SYBR Green chemistry. Relative mRNA expression levels were normalized to GAPDH or ACTB and calculated using the 2^−ΔΔCt^ method. Primer sequences are listed in Table S1.

### 2.9 Statistical Analysis

Statistical analyses were performed using Prism version 8.4.3 (GraphPad Software, San Diego, CA, USA). Data are presented as mean ± SD. The normality of each dataset within each group was assessed using the Shapiro–Wilk test. For comparisons between two independent groups, an unpaired two‐tailed Student's *t*‐test was used when data in both groups conformed to a normal distribution. If either group failed the normality test, the Mann–Whitney *U* test was applied. For time‐course data involving group and age as factors, two‐way ANOVA was performed, followed by Holm‐Šídák post hoc multiple comparisons when significant main effects or interactions were identified. Statistical significance was defined as *p* < 0.05.

## 3 Results

### 3.1 Sustained Hypertension Induces Early Cerebrovascular Alterations, Demyelination, and Later Behavioral Decline

**FIGURE 1:**
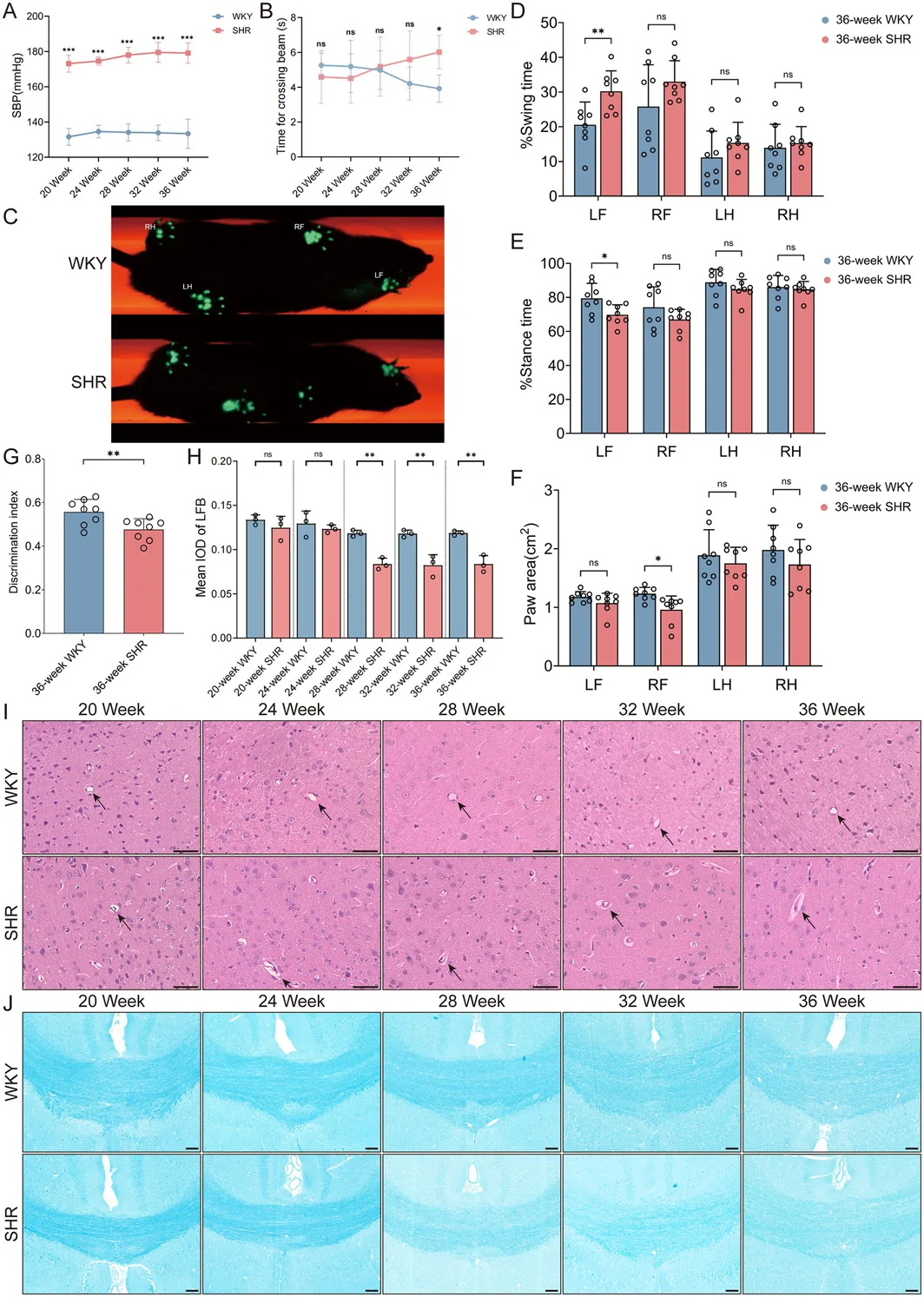
Sustained hypertension induces progressive behavioral deficits, cerebrovascular injury, and demyelination in SHRs. (A) Systolic blood pressure (SBP) in SHRs and WKY rats from 20 to 36 weeks of age (*n* = 8 per group). (B) Beam‐walking test: Time required to cross the beam from 20 to 36 weeks of age (*n* = 7 per group). (C) Representative ventral‐view images from CatWalk XT videos showing gait patterns. (D–F) Quantitative analysis of swing time (%), stance time (%), and paw area, respectively (*n* = 8 per group). (G) Discrimination index in the novel object recognition test at 36 weeks (*n* = 8 per group). (H) Quantitative analysis of Luxol Fast Blue (LFB) from 20 to 36 weeks of age (*n* = 3 per group). (I) Representative hematoxylin and eosin (HE) staining of deep cortical vessels. Black arrows point to small blood vessels. Scale bar = 50 μm. (J) Representative LFB staining of the corpus callosum. Scale bar = 50 μm. Data are presented as mean ± SD. ns, not significant; *p < 0.05, **p < 0.01, ***p < 0.001 compared with the WKY group. LF, left forelimb; LH, left hindlimb; RF, right forelimb; RH, right hindlimb.

SHRs exhibited persistently elevated systolic blood pressure throughout the observation period, confirming a sustained hypertensive state (Figure 1A). Beam‐walking performance worsened from 28 weeks and reached statistical significance at 36 weeks, indicating impaired fine motor coordination at the stage (Figure 1B). Quantitative gait analysis showed prolonged swing time percentage and shortened stance time percentage in the left forelimb, along with a reduced paw print area in the right forelimb (Figure 1C–F). Nonspatial recognition memory, assessed using the novel object recognition test, was also impaired in SHRs at 36 weeks, as evidenced by a significantly lower discrimination index compared with WKY rats (Figure 1G). In parallel, LFB staining showed preserved myelin at 20 and 24 weeks but significant myelin loss in the corpus callosum from 28 weeks onward (Figure 1H,J). HE staining suggested early vascular morphological abnormalities in SHRs, including irregular vessel profiles and enlarged perivascular spaces from 20 weeks onward (Figure 1I). Together, these findings indicate that sustained hypertension in SHRs leads to early cerebrovascular injury, followed by progressive demyelination and delayed functional decline.

### 3.2 Longitudinal DTI Delineates 28 Weeks as the Onset of WMLs Preceding Brain Atrophy

**FIGURE 2:**
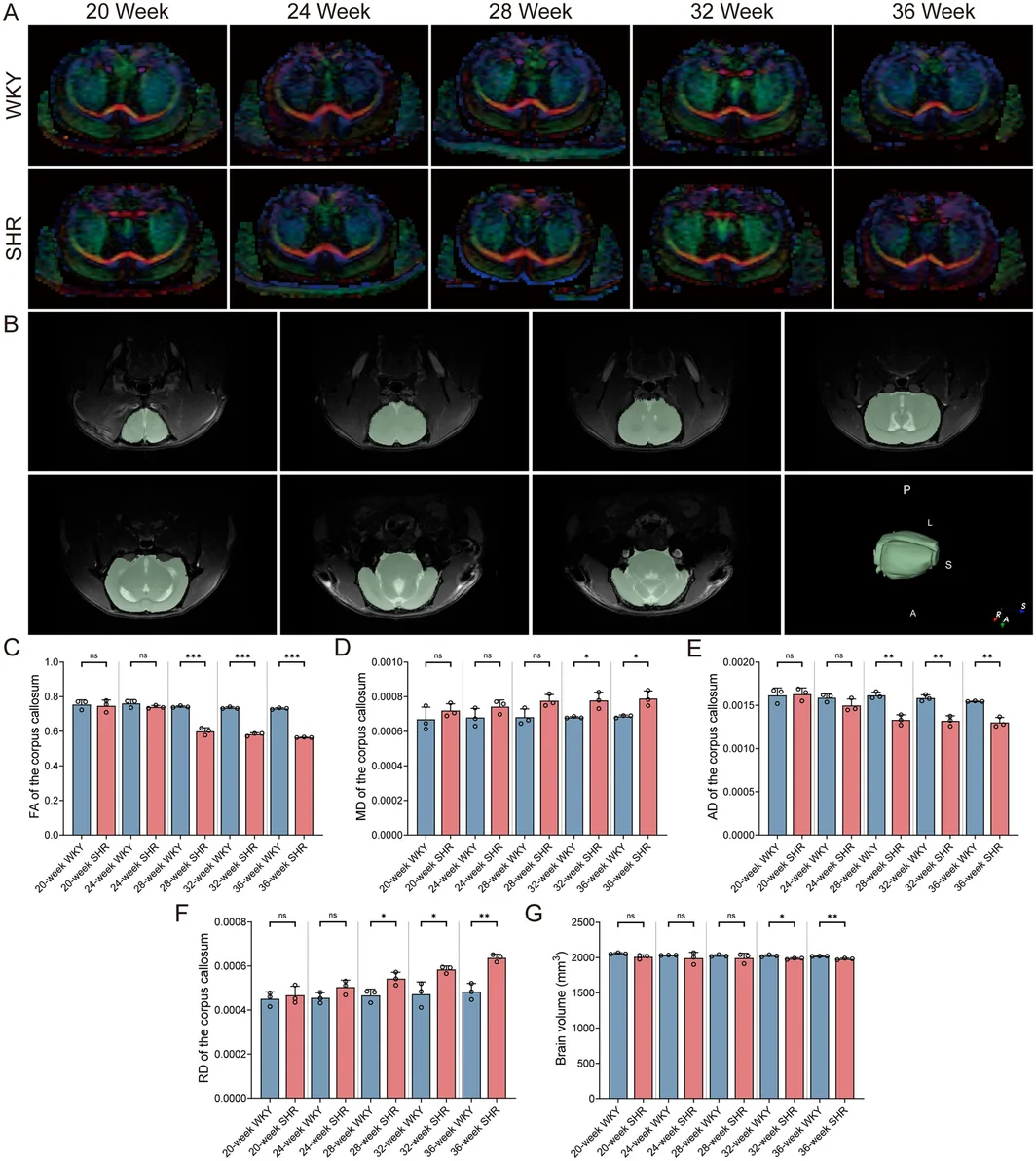
Longitudinal MRI reveals the onset and progression of white matter injury and brain atrophy in SHRs. (A) Representative color‐coded FA images of the corpus callosum from 20 to 36 weeks of age. (B) Representative T2‐weighted brain image masking and 3D surface reconstruction for volumetric analysis. (C–F) Quantitative analysis of FA, MD, AD, and RD values from 20 to 36 weeks of age (*n* = 3 per group). (G) Quantitative analysis of total brain volume from 20 to 36 weeks of age (*n* = 3 per group). Data are presented as mean ± SD. ns, not significant; *p < 0.05, **p < 0.01, ***p < 0.001 compared to the WKY group. AD, axial diffusivity; FA, fractional anisotropy; MD, mean diffusivity; RD, radial diffusivity; A, anterior; L, left; P, posterior; R, right; S, superior.

Longitudinal MRI further defined the temporal onset of white matter injury in SHRs. Representative FA maps from 20 to 36 weeks are shown (Figure 2A). The process for constructing 3D brain surface models for volumetric measurement is illustrated (Figure 2B). DTI showed no significant differences between SHRs and WKY rats at 20 or 24 weeks. From 28 weeks onward, SHRs exhibited reduced FA and increased RD in the corpus callosum, accompanied by a decline in AD. MD increased later, from 32 weeks onward, consistent with progressive disruption of tissue integrity (Figure 2C–F). Brain volumetry revealed no difference between groups up to 28 weeks, whereas total brain volume was significantly reduced in SHRs at 32 and 36 weeks (Figure 2G). These results suggest that 28 weeks represents the onset of detectable white matter microstructural impairment in SHR, preceding overt brain atrophy.

### 3.3 Histopathological Validation Confirms Demyelination and Altered Oligodendroglial Lineage Markers at the Critical 28‐Week Time Point

**FIGURE 3:**
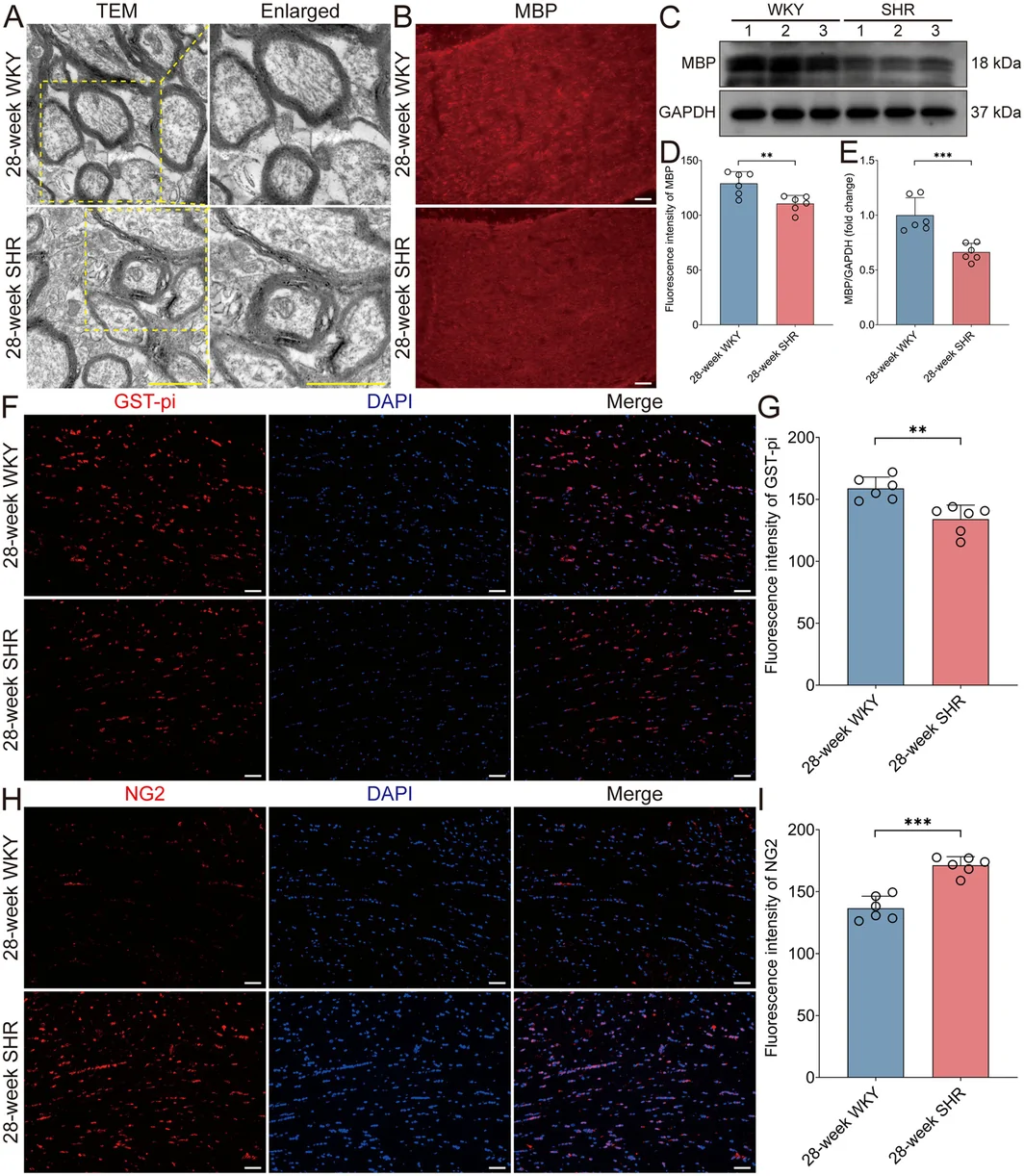
Histopathological validation confirms demyelination and altered oligodendroglial lineage‐associated markers at the critical 28‐week time point in SHRs. (A) Representative transmission electron microscope (TEM) images showing the ultrastructure of myelin sheaths and myelinated axons in the corpus callosum. Enlarged panels on the right highlight myelin lamellar disruption and axonal degeneration. Scale bar = 1 μm. (B) Representative immunofluorescence images of MBP (red) in the corpus callosum. Scale bar = 50 μm. (C) Western blot of MBP expression in the corpus callosum of WKY and SHR groups. (D) Quantitative analysis of MBP fluorescence intensity (*n* = 6 per group). (E) Quantitative analysis of MBP protein levels normalized to GAPDH (*n* = 6 per group). (F) Representative immunofluorescence images of GST‐pi‐positive mature oligodendrocytes. Scale bar = 50 μm. (G) Quantitative analysis of GST‐pi fluorescence intensity (*n* = 6 per group). (H) Representative immunofluorescence images of NG2‐positive oligodendrocyte precursor cells. Scale bar = 50 μm. (I) Quantitative analysis of NG2 fluorescence intensity (*n* = 6 per group). Data are presented as means ± SD. **p < 0.01, ***p < 0.001 compared to the WKY group. MBP, myelin basic protein; TEM, transmission electron microscopy.

Based on the MRI‐defined onset window, we further characterized pathological alterations in 28‐week‐old SHRs. Transmission electron microscopy revealed characteristic myelin abnormalities, including lamellar loosening, focal splitting, disruption, and blurred axonal contours in the corpus callosum of SHRs (Figure 3A). MBP, a key structural component of the myelin sheath [26], exhibited significantly reduced levels in both the immunofluorescence staining and western blot (Figure 3B–E). SHRs also showed decreased GST‐pi fluorescence intensity and increased NG2 fluorescence intensity (Figure 3F–I), indicating altered mature oligodendrocyte (OL)‐ and oligodendrocyte precursor cell (OPC)‐associated marker profiles at the 28‐week stage.

### 3.4 Neurovascular Dysfunction and Inflammation Converge at 28 Weeks

**FIGURE 4:**
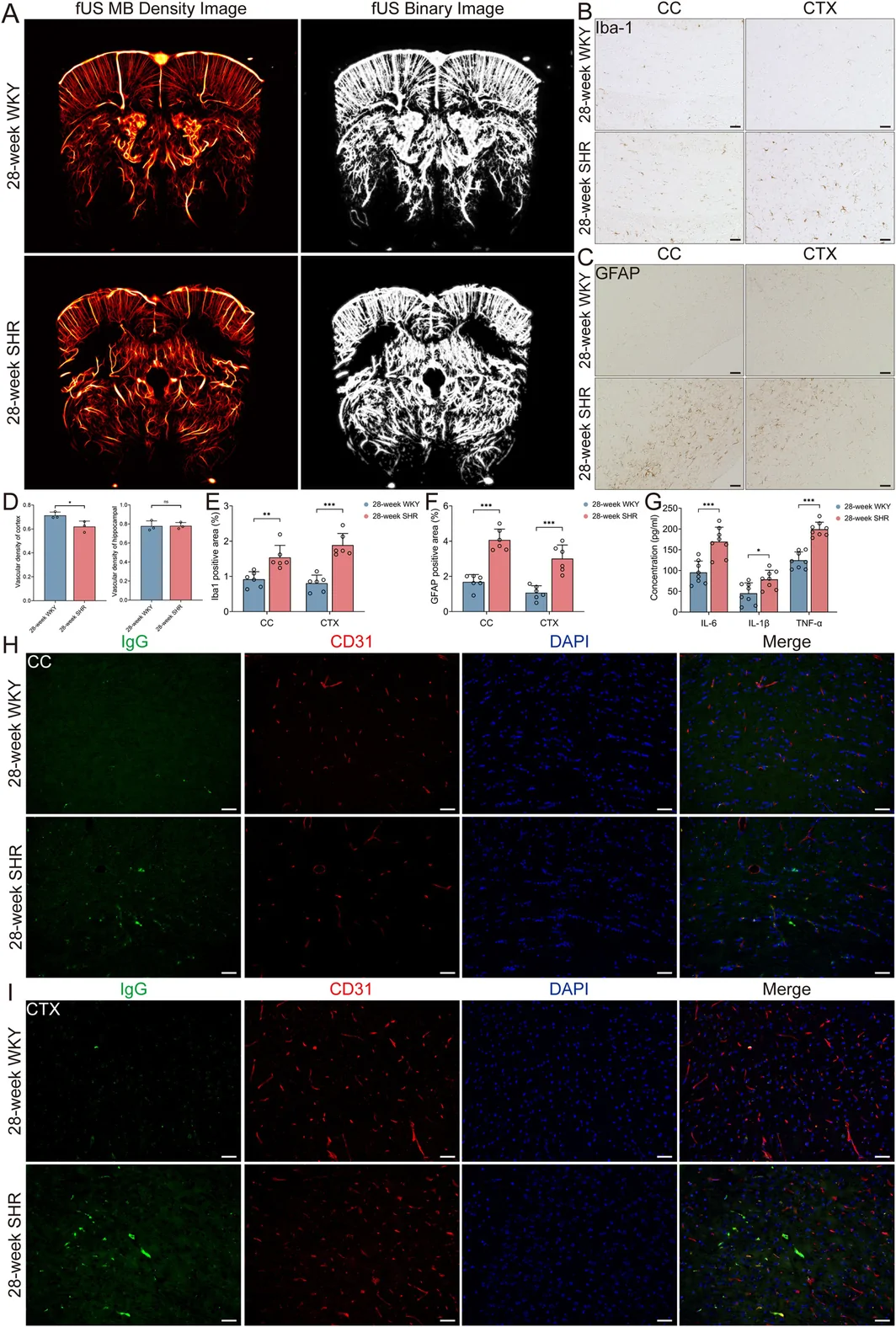
Convergent neurovascular dysfunction and inflammation at the critical 28‐week time point in SHRs. (A) Representative super‐resolved cerebral microvascular maps acquired by functional ultrasound (FUS) localization microscopy and corresponding binarized images. (B, C) Immunohistochemical staining for Iba1 and GFAP in the corpus callosum (CC) and cortex (CTX), respectively. Scale bar = 50 μm. (D) Quantitative analysis of cortical and hippocampal microvascular density obtained from FUS imaging (*n* = 3 per group). (E, F) Quantification of Iba1‐ and GFAP‐positive areas in the CC and CTX, respectively (*n* = 6 per group). (G) Serum concentrations of IL‐6, IL‐1β, and TNF‐α (*n* = 8 per group). (H, I) Representative immunofluorescence co‐staining for CD31 and endogenous IgG in the CC and CTX, respectively. Scale bar = 50 μm. Data are presented as mean ± SD. ns, not significant; *p < 0.05, **p < 0.01, ***p < 0.001 compared to the WKY group. CC, corpus callosum; CTX, cortex; FUS, functional ultrasound; MB, microbubble.

ULM revealed extensive rarefaction and disorganization of cortical microvessels in SHRs compared with WKY rats (Figure 4A). Representative immunohistochemical staining showed Iba1‐ and GFAP‐positive immunoreactivity in the corpus callosum and cortex (Figure 4B,C). Quantitative analysis confirmed a significant reduction in cortical microvessel density, whereas hippocampal microvascular density showed no notable difference (Figure 4D). Inflammatory activation was evidenced by increased Iba1‐ and GFAP‐positive areas within the corpus callosum and cortex of SHRs and elevated serum IL‐6, IL‐1β, and TNF‐α concentrations (Figure 4E–G). CD31 and endogenous IgG co‐immunostaining was performed to assess vascular integrity and BBB permeability. In WKY rats, IgG signals were largely confined within or close to CD31‐positive vascular structures. In contrast, SHRs showed discontinuous CD31‐positive vascular profiles accompanied by increased perivascular and parenchymal IgG signals, indicating IgG extravasation and increased BBB permeability (Figure 4H,I).

### 3.5 Transcriptomics Unveils cAMP Signaling in WMLs of CSVD

Transcriptomic analysis of the corpus callosum was performed to elucidate molecular mechanisms underlying WMLs of CSVD in SHRs. Principal component analysis (PCA) revealed clear separation between SHRs and WKY controls (Figure S1A). Differential expression analysis identified 339 DEGs, including 215 upregulated and 124 downregulated genes (Figure S1B). The full list of significantly differentially expressed genes is provided in Supporting Information S1. Gene Ontology (GO) enrichment showed biological processes linked to vascular function, neurotransmission, and immune response (Figure S1C). Cellular component analysis revealed enrichment in cholinergic synapse, neuronal dense core vesicle, axon, and dendrite (Figure S1D). Molecular function analysis highlighted sodium and calcium channel activities and acetylcholine receptor activity (Figure S1E). Notably, Kyoto Encyclopedia of Genes and Genomes (KEGG) analysis identified significant enrichment of the cAMP signaling pathway (Figure S1F), which was further confirmed by gene set enrichment analysis (GSEA) (Figure S1G). These findings suggest that cAMP‐related signaling may contribute to WML pathogenesis in SHRs. This pathway may regulate neural and vascular functions through downstream effectors such as PKA and CREB, thereby contributing to cerebrovascular disease progression [27, 28].

### 3.6 Proteomic Profiling Highlights Pde10a as a Candidate Associated With cAMP Signaling in WMLs of CSVD

Proteomic analysis of the corpus callosum further characterized protein alterations in SHRs. Quality control assessments confirmed high data reliability for subsequent analyses (Figure S2). PCA demonstrated clear separation between groups, and differential expression analysis identified 95 DEPs, including 57 upregulated and 38 downregulated proteins (Figure S3A–D). The full list of significantly differentially expressed proteins is provided in Supporting Information S2. GO enrichment analysis showed significant enrichment of DEPs in biological processes related to vascular function, immune response, and neurotransmission (Figure S3E). KEGG pathway analysis revealed that these proteins primarily participate in metabolic, immune, and neural function pathways (Figure S3F,G). Protein–protein interaction (PPI) network analysis further illustrated functional interactions among DEPs (Figure S3H). Among all DEPs, Pde10a exhibited striking expression change in SHRs, underscoring its potential as a key molecular node in WMLs. To further define functional distinctions among DEPs, we stratified them into four fold‐change intervals (Q1–Q4) based on fold‐change magnitude (Figure S3I). Biological processes of GO analysis revealed that DEPs in SHRs were broadly associated with neuroinflammation, endothelial dysfunction, and sodium/calcium channel activities (Figure S3J). Notably, proteins in Q4 (fold change most significantly altered) were strongly enriched in the cAMP signaling pathway, indicating the crucial involvement in WMLs development (Figure S3K). Integrative KEGG, GO, and PPI analyses consistently implicated Pde10a as a central mediator of cAMP signaling, linking neuroinflammatory and vascular regulatory mechanisms. Prior studies further support Pde10a's roles in neurodegeneration, cognitive dysfunction, and vascular pathology, reinforcing its potential significance in WML pathogenesis [29, 30, 31].

### 3.7 Integrated Transcriptomic and Proteomic Analyses Highlight cAMP Pathway Dysregulation

**FIGURE 5:**
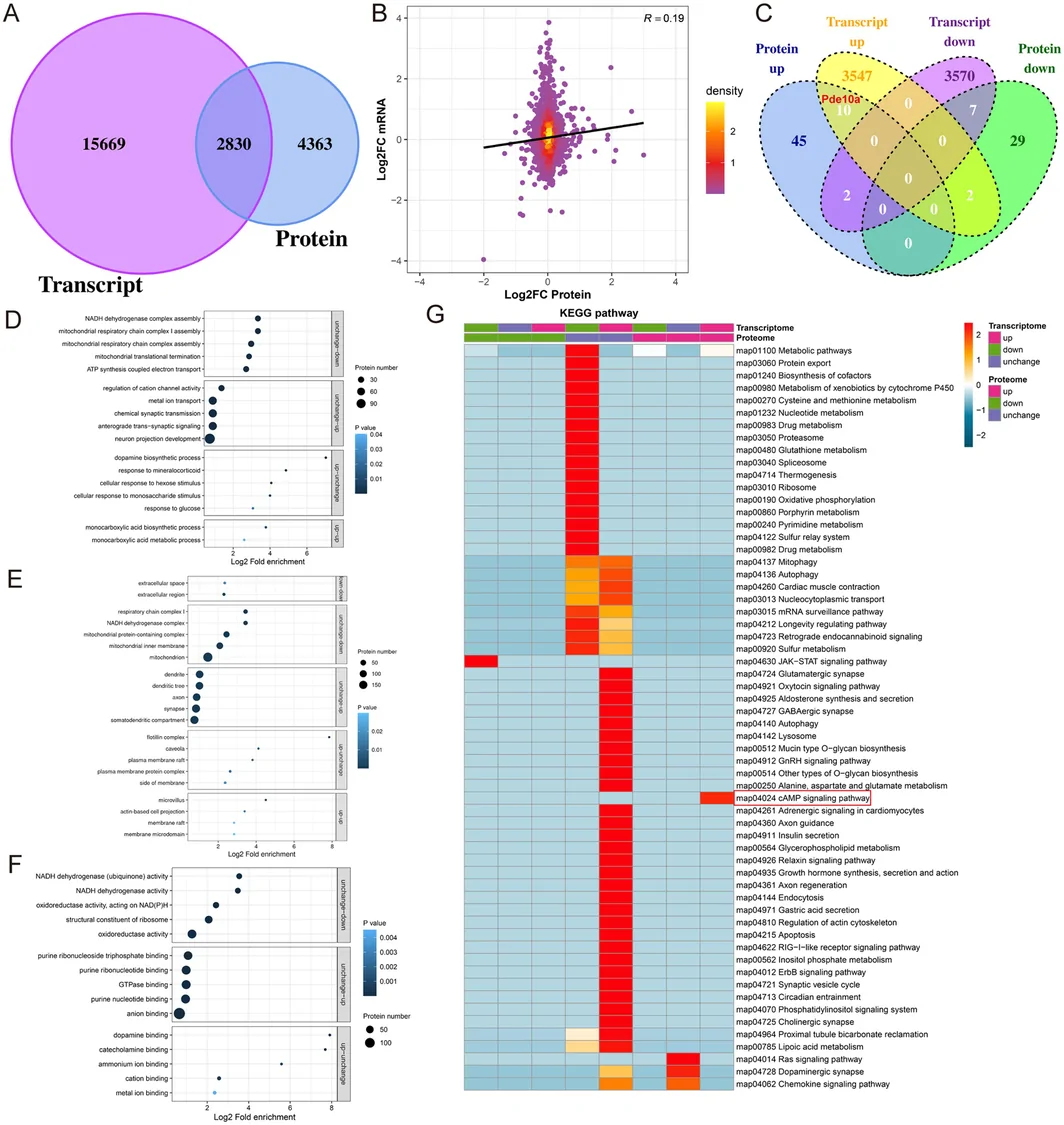
Integrated transcriptomic and proteomic analyses highlight cAMP pathway dysregulation in SHR‐related WMLs. (A) Venn diagram depicting all quantified molecules from transcriptomic and proteomic analyses, identifying 2830 genes consistently detected at both transcript and protein levels. (B) Scatter plot illustrating a positive correlation between mRNA abundance and corresponding protein expression. (C) Venn diagram showing the concordance between transcriptomic and proteomic expression profiles. (D–F) GO enrichment analysis of molecules in transcript and protein levels was integrated, significantly enriched GO terms in (D) biological processes, (E) cellular components, and (F) molecular functions. (G) KEGG pathway enrichment analysis in distinct regulatory patterns between transcript and protein levels. *n* = 6 per group.

To identify convergent molecular changes, we integrated the transcriptomic and proteomic datasets. The analysis quantified 18,499 transcripts and 7193 proteins. Comparative evaluation identified 2830 genes consistently detected at both transcript and protein levels (Figure 5A). Transcript and protein abundances showed an overall positive correlation (Figure 5B). Among concordantly regulated molecules, Pde10a showed an upward trend at the transcript level and was significantly upregulated at the proteomic level (Figure 5C). Functional enrichment of the integrated dataset revealed strong associations with neuroinflammatory signaling, vascular injury, and the cAMP signaling pathway, particularly among molecules upregulated in both omics layers (Figure 5D–G). Collectively, these analyses identify cAMP pathway dysregulation as a convergent molecular feature and support Pde10a as a proteomics‐driven candidate associated with WML development in SHRs.

### 3.8 Increased Pde10a With Decreased Downstream cAMP/PKA/CREB Expression in SHRs

**FIGURE 6:**
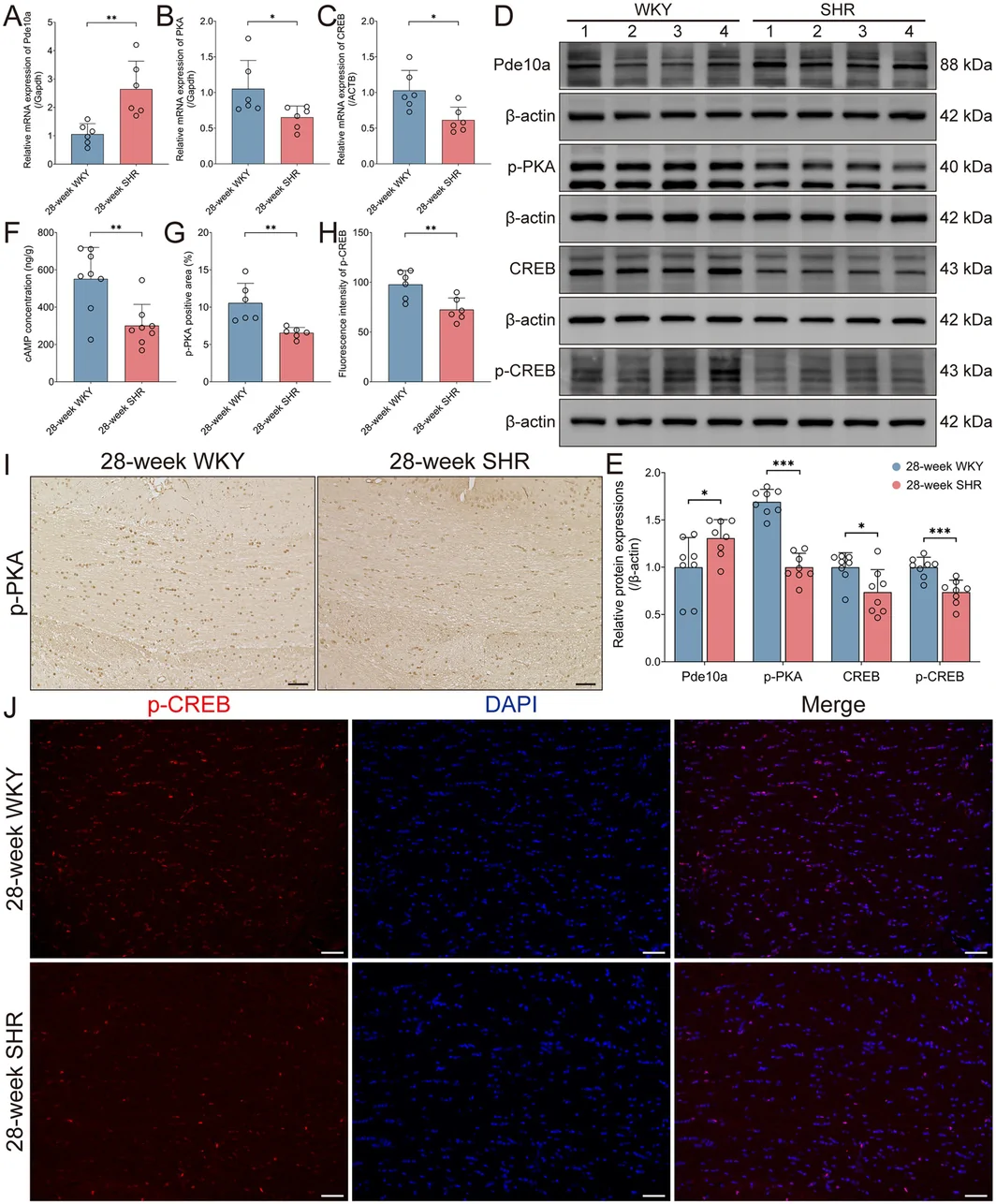
Increased Pde10a with decreased downstream cAMP/PKA/CREB expression in 28‐week‐old SHRs. (A–C) mRNA expression levels of Pde10a, PKA, and CREB measured by RT‐qPCR (*n* = 6 per group). (D) Representative western blots showing Pde10a, p‐PKA, CREB, and p‐CREB expression in the corpus callosum (CC) of WKY and SHR groups. (E) Quantitative analysis of Pde10a, p‐PKA, CREB, and p‐CREB levels normalized to β‐Actin (*n* = 8 per group). (F) cAMP concentration in corpus callosum determined by ELISA (*n* = 8 per group). (G) Quantification of p‐PKA‐positive area in the CC (*n* = 6 per group). (H) Quantitative analysis of p‐CREB fluorescence intensity in the CC (*n* = 6 per group). (I) Immunohistochemistry for p‐PKA in the CC. Scale bar = 50 μm. (J) Representative immunofluorescence images of p‐CREB in the CC. Scale bar = 50 μm. Data are presented as mean ± SD. *p < 0.05, **p < 0.01, ***p < 0.001 compared to the WKY group.

To further validate the Pde10a/cAMP pathway, we examined Pde10a and its downstream effectors in the corpus callosum of 28‐week‐old SHRs. Although Pde10a did not reach significance in the RNA‐seq dataset, RT‐qPCR showed increased Pde10a mRNA expression together with reduced PKA and CREB mRNA levels (Figure 6A–C). Western blot analysis confirmed upregulation of Pde10a protein and decreases in p‐PKA, CREB, and p‐CREB in SHRs (Figure 6D,E). ELISA further demonstrated reduced cAMP concentrations in SHR brain tissue (Figure 6F). Consistently, immunohistochemistry and immunofluorescence showed reduced p‐PKA‐positive area and lower p‐CREB fluorescence intensity in the corpus callosum of SHRs (Figure 6G–J). These findings confirm marked suppression of downstream cAMP/PKA/CREB signaling in association with Pde10a upregulation in SHRs.

## 4 Discussion

In this study, serial 9.4‐T MRI‐DTI enabled in vivo tracking of white matter microstructural alterations combined with pathological analyses identified 28 weeks of age as a critical window for the onset of WMLs in SHRs; T2‐weighted volumetric analysis captured later brain atrophy. In addition, ULM provided high‐resolution visualization of cerebral microvascular rarefaction at the critical 28‐week time point. Together with behavioral assessments, these multimodal approaches strengthened the temporal characterization of WML progression and associated neurovascular dysfunction [32].

Hypertension‐induced chronic hypoperfusion and BBB dysfunction are recognized contributors to CSVD [13]. Neuroimaging studies have demonstrated increased BBB permeability in white matter hyperintensity regions of patients with CSVD, while postmortem analyses revealed upregulation of ischemia‐hypoxia markers in the same regions [33, 34]. Chronic hypoperfusion driven by hypertension induces myelin degradation, axonal loss, and focal BBB impairment [35, 36]. Local BBB breakdown subsequently permits the entry of circulating plasma constituents into the central nervous system, triggering vascular wall injury and diffuse myelin and axonal damage within the white matter [37, 38]. Prior work in SHRs reported BBB leakage in deep cortical regions by 24 weeks and demyelination with brain atrophy at later stages [16]. Our findings extend these observations by defining the dynamic progression more precisely and identifying 28 weeks as the transition point at which white matter injury becomes clearly detectable.

At this critical time point, sustained hypertension had already induced small‐vessel morphological alterations, whereas overt demyelination in the corpus callosum emerged at 28 weeks and was confirmed by DTI, LFB staining, and MBP reduction. Behavioral deficits appeared later, indicating that structural white matter injury precedes functional decline and may therefore represent an early experimental window for investigating mechanisms associated with WML. Notably, at 28 weeks, SHRs exhibited reduced GST‐pi‐positive mature OL signals and increased NG2‐positive OPC signals. When myelin sheath changes, the differentiation of OPCs into mature OLs was blocked, and axonal degeneration and neuronal apoptosis were induced [39]. The recruitment and differentiation of OPCs to OLs are essential for axonal ensheathment and myelin sheath reconstruction during repair and regeneration [40, 41]. Our findings preliminarily suggest altered oligodendroglial lineage‐associated profiles in SHRs. These changes may indicate a delay in OPC maturation or an imbalance in oligodendrocyte turnover, which could be associated with insufficient myelin maintenance during WML progression.

Neurovascular dysfunction and inflammation also converged at 28 weeks. ULM imaging demonstrated reduced cortical microvascular density, while CD31/IgG staining revealed endothelial disruption and BBB leakage in the corpus callosum and deep cortex. These findings are consistent with those of Fu et al., who reported reduced MBP expression and IgG leakage in the deep white matter of 28‐week‐old SHRs, corroborating CSVD‐related phenotypes [42]. Correspondingly, these changes were accompanied by increased microglial and astrocytic activation and elevated circulating IL‐1β, IL‐6, and TNF‐α, indicating coexisting central and peripheral inflammatory responses. This finding aligns with previous evidence that hypertension induces a systemic pro‐inflammatory bias in SHRs [43]. Elevated circulating cytokines in 28‐week‐old SHRs suggest that systemic inflammatory priming may represent an additional pathobiological background that cooperates with BBB disruption and glial activation to promote WML progression. Activated microglia can release pro‐inflammatory mediators that impair OPC survival and differentiation, thereby contributing to disrupted oligodendroglial maturation and myelin injury [44]. Moreover, persistent neuroinflammation further impairs vascular function and BBB stability, collectively aggravating myelin injury [45, 46].

To explore potential molecular drivers, we integrated transcriptomic and proteomic data from the corpus callosum at the 28‐week stage. Transcriptomic enrichment analysis highlighted dysregulation of the cAMP signaling pathway, whereas proteomic analysis identified Pde10a as a significantly upregulated protein in SHRs. Pde10a is predominantly enriched in neuronal populations within the central nervous system, where it regulates intracellular cyclic nucleotide signaling by hydrolyzing the second messenger cAMP [47, 48]. This observation was further validated experimentally. RT‐qPCR and western blot confirmed elevated Pde10a expression at both mRNA and protein levels in SHRs, while ELISA revealed a pronounced reduction in brain cAMP concentration and suppression of downstream signaling, as reflected by decreased levels of p‐PKA and p‐CREB. The Pde10a/cAMP/PKA/CREB cascade plays a vital role in OLs' survival, OPCs' differentiation, vascular homeostasis, and inflammatory responses. On the one hand, Pde10a is critical in OPCs' regulation, in the ethidium bromide induced demyelination model, Pde10a expression was increased, whereas treatment with a Pde10a inhibitor promoted cAMP signaling, enhanced primary OPCs' differentiation and maturation, and subsequently reversed demyelination and motor deficits [49]. In vascular endothelial cells, the cAMP/PKA axis maintains BBB integrity and modulates vascular tone. The cAMP activation of PKA enhances endothelial nitric oxide synthase activity, promoting nitric oxide mediated vasodilation and supporting endothelial health [50]. Moreover, cAMP/PKA signaling stabilizes endothelial tight junctions, thereby preserving BBB function [51, 52]. In addition, cAMP/PKA signaling exerts potent anti‐inflammatory effects in microglia, promoting their polarization toward anti‐inflammatory phenotypes [53, 54]. The downstream p‐CREB also has been related to reductions in neuroinflammation [55]. Activated p‐CREB interferes with nuclear factor kappa B (NF‐κB) signaling by dissociating CREB‐NF‐κB complexes, thereby inhibiting transcription of proinflammatory mediators such as IL‐6 and TNF‐α [56]. Therefore, Pde10a upregulation accompanied by suppression of downstream cAMP/PKA/CREB signaling may represent a shared mechanism connecting demyelination, vascular dysfunction, and neuroinflammation in hypertension‐related WMLs. And targeting this pathway may offer a promising therapeutic strategy for white‐matter protection.

While this study delineated the progression and molecular mechanisms underlying WMLs in SHRs, several limitations should be acknowledged. First, only male rats were used in this exploratory time‐course study, limiting the assessment of potential sex differences in CSVD progression. Second, because the corpus callosum is the largest and most reproducible white matter structure in rats, it was selected as the primary region for assessing WMLs; however, corpus callosum injury in SHRs should be interpreted as an experimental indicator of hypertension‐associated white matter vulnerability rather than a complete anatomical equivalent of human CSVD‐related WMLs. Third, although Pde10a/cAMP signaling dysregulation was identified, its cell‐type‐specific roles and causal contribution require further validation using targeted genetic or pharmacological approaches.

## 5 Conclusion

In conclusion, this study identified 28 weeks of age as the critical onset window for hypertension‐induced WMLs in SHRs and revealed concurrent neurovascular dysfunction and inflammatory activation at this stage. Dysregulation of the Pde10a/cAMP signaling pathway may represent a candidate molecular mechanism associated with WML development. These findings advance our understanding of CSVD pathophysiology and support further investigation of Pde10a‐associated cAMP signaling as a potential therapeutic target for white matter protection in CSVD.

## Funding

This work was supported by the Beijing University of Chinese Medicine “Decoding Chinese Medicine” collaborative project (BZY‐JMZY‐2022‐002).

## Ethics Statement

All experimental protocols were approved by the Experimental Animal Center of Beijing University of Chinese Medicine (Approval No. BUCM‐2024053104‐2226). All animal procedures were conducted in accordance with the ARRIVE guidelines.

## Conflicts of Interest

The authors declare no conflicts of interest.

## Supporting information


**Table S1:** Primer sequence used for RT‐qPCR.
**Figure S1:** Transcriptomic profiling reveals cAMP signaling enrichment in white matter lesions of CSVD. (A) Principal component analysis (PCA) of transcriptomic data demonstrates clear segregation between SHRs and WKY groups. (B) Volcano plot depicting differentially expressed genes (DEGs) between SHRs and WKY groups; red indicates upregulated genes, blue indicates downregulated genes, and gray indicates non‐significant genes. (C–E) Gene Ontology (GO) enrichment analysis of DEGs showing significantly enriched (C) biological processes, (D) cellular components, and (E) molecular functions. (F) Kyoto Encyclopedia of Genes and Genomes (KEGG) pathway enrichment highlights pathways related to neurodegeneration and vascular dysfunction. (G) Gene set enrichment analysis (GSEA) confirms significant enrichment of the cAMP signaling pathway in SHRs. The green line represents the enrichment score (ES), with the Y‐axis peak indicating the maximum ES value. *n* = 6 per group.
**Figure S2:** Quality control and proteomic profiling of corpus callosum in SHR and WKY groups. (A) Total identified peptides, unique peptides, identified proteins, and comparable proteins in the proteomic analysis. (B) Quantifiable proteins and their cumulative distribution across WKY and SHR samples. (C) Log10 intensity distribution for the proteome across samples, with intensity categories highlighted by different colors, reflecting the protein abundance in each sample. *n* = 6 per group.
**Figure S3:** Proteomic profiling highlights Pde10a as an upregulated candidate associated with cAMP signaling in CSVD‐related WMLs. (A) PCA of proteomic data demonstrates clear segregation between SHR and WKY groups. (B) Bar plot summarizing differentially expressed proteins (DEPs): 57 upregulated (red) and 38 downregulated (blue). (C) Volcano plot showing DEPs between SHRs and WKY groups; red indicates significantly upregulated proteins, blue indicates downregulated proteins, and gray indicates non‐significant proteins. (D) Heatmap of all identified DEPs illustrating intergroup clustering. (E) GO enrichment of DEPs in biological process categories. (F) KEGG pathway enrichment highlighting metabolic, immune, and neural signaling pathways. (G) Chord diagram illustrating the relationships between DEPs and enriched KEGG pathways. (H) Protein–protein interaction (PPI) network of DEPs; red nodes represent upregulated proteins, blue nodes represent downregulated proteins, and node size correlates with interaction degree. (I) Stratification of DEPs into four fold‐change intervals (Q1–Q4) based on fold‐change magnitude, from lowest (Q1) to highest (Q4). (J) Biological processes of GO enrichment terms associated with DEPs across intervals. (K) KEGG enrichment for Q4 proteins showing marked enrichment of the cAMP signaling pathway. *n* = 6 per group.

**Data S1:** Full list of significantly differentially expressed genes.

**Data S2:** Full list of significantly differentially expressed proteins.

## Data Availability

The data that support the findings of this study are available from the corresponding author upon reasonable request.
