# Supplementary material for: Temporal Mapping of CSVD‐Related White Matter Lesions and Concurrent Neurovascular Dysfunction in Spontaneously Hypertensive Rats
Source: CNS Neurosci Ther. 2026 Sep 11;32(9):e71142. doi: 10.1002/cns.71142 (PMC13563166; doi:10.1002/cns.71142)
Supplement: Supplementary file 1 [file CNS-32-e71142-s002.docx]

**Temporal Mapping of CSVD-Related White Matter Lesions and Concurrent Neurovascular Dysfunction in Spontaneously Hypertensive Rats**

Jing Peng^1,2^, Jingnian Ni^1,2^, Fuyao Li^2,3^, Ting Li^1,2^, Mingqing Wei^1,2^, Tianci Ge^1,2^, Lulu Yao^1,2^, Jing Shi^1,2,*^, Jinzhou Tian^1,2,*^

^1^Department of Neurology, Dongzhimen Hospital, Beijing University of Chinese Medicine, Beijing 100700, China

^2^Institute for Brain and Cognitive Disorders, Beijing University of Chinese Medicine, Beijing 100029, China

^3^Institute of Traditional Chinese Medicine/National Key Laboratory, Beijing University of Chinese Medicine, Beijing 100029, China

**^*^Correspondence:** jztian@hotmail.com (J.Z. Tian); [sshijing87@163.com](mailto:sshijing87@163.com) (J. Shi)

**Supplementary Methods**

**Animals and Study Design**

At each scheduled time point (20, 24, 28, 32, and 36 weeks), animals were selected from the available pool using a random-number table before outcome assessment. Systolic blood pressure was measured in 8 rats per group, beam-walking performance was assessed in 7 rats per group, and MRI was performed in 3 rats per group. Histological assessments of vascular morphology and myelin integrity, including HE and LFB staining, were performed in corresponding terminal subsets, with LFB quantification performed in 3 rats per group at each time point. At 36 weeks, CatWalk XT gait analysis and novel object recognition testing were performed in 8 rats per group.

At the identified critical 28-week time point, additional mechanistic analyses were performed using randomly selected animals according to assay requirements. Ultrasound localization microscopy was performed in 3 rats per group; immunofluorescence or immunohistochemical analyses of myelin, oligodendroglial, and inflammatory markers were performed in 6 rats per group; ELISA and western blot analyses were performed in 8 rats per group unless otherwise indicated; and transcriptomic and proteomic analyses were performed in 6 rats per group. The animal selection was performed before data acquisition and was not based on behavioral, imaging, histological, or molecular outcomes.

**MRI Acquisition and Analysis**

MRI was performed using a 9.4-Tesla animal MRI scanner (Bruker BioSpec) at the Tsinghua Laboratory of Brain and Intelligence. Rats were anesthetized with 3–4% isoflurane for induction and maintained under 2% isoflurane throughout imaging, with oxygen flow regulated at 0.8 L/min to ensure stable respiration and physiological homeostasis.

T2-weighted images were acquired using a turbo spin-echo rapid acquisition with relaxation enhancement (RARE) sequence with the following parameters: TR/TE = 3673/33 ms, field of view (FOV) = 35 mm × 35 mm, slice thickness = 0.5 mm, 35 slices, matrix size = 256 × 256, and 3 averages. Diffusion-weighted images were acquired using an echo-planar imaging (EPI) sequence to assess white matter microstructural alterations with the following parameters: TR/TE = 2000/22 ms, FOV = 20 mm × 18 mm, slice thickness = 0.7 mm, 35 slices, matrix size = 80 × 72, and 3 averages. DTI were acquired using a single-shell protocol with 30 non-collinear diffusion-encoding directions at a b-value of 1000 s/mm^2^, together with 5 non-diffusion-weighted images (b = 0 s/mm²).

For brain volumetric reconstruction, T2-weighted images were imported into 3D Slicer software. Brain boundaries were manually delineated slice by slice on coronal images using the Segment Editor module by an investigator blinded to group identity. After all slices covering the brain were segmented, the “Fill between slices” function was applied to interpolate the segmented regions and generate a continuous three-dimensional brain mask. A 3D surface model was then reconstructed from this mask, and total brain volume was calculated using the Segment Statistics module. For DTI analysis, fractional anisotropy (FA), mean diffusivity (MD), axial diffusivity (AD), and radial diffusivity (RD) were computed in regions of interest (ROIs) within the corpus callosum to assess white matter microstructure. FA quantifies the directional coherence of water diffusion (range: 0–1), with lower values reflecting disrupted fiber organization [S1]. MD represents the mean water diffusivity across all directions, with higher values indicating increased overall water mobility within tissue [S2]. RD measures diffusion perpendicular to fiber tracts and is often associated with changes in myelin integrity [S3]. AD reflects water diffusion parallel to the principal fiber axis, and decreased AD may be associated with axonal alterations or injury [S4]. Color-coded FA maps were generated by combining the principal eigenvector orientation with the FA map to produce RGB representations of fiber directionality.

**Luxol Fast Blue Staining**

Following cardiac perfusion with phosphate-buffered saline (PBS) and 4 % paraformaldehyde (PFA), whole brains were carefully extracted, dehydrated, embedded in paraffin, and coronally sectioned at 4 μm thickness using a microtome (Leica, Germany). For Luxol Fast Blue (LFB) staining, paraffin sections were thoroughly deparaffinized and rehydrated in distilled water. Sections were then immersed in pre-warmed LFB staining solution (65 °C) for 4 h under a sealing film to prevent evaporation. After rinsing under running tap water, differentiation was performed in 0.05 % lithium carbonate for 3–5 s, followed by 70 % ethanol for 5-10 s, until optimal contrast between blue-stained myelinated white matter and the pale background was achieved. Finally, sections were dehydrated, cleared, and coverslipped. Stained sections were examined and imaged using a KEYENCE BZ-X810 bright-field microscope (Keyence Corp., Osaka, Japan) under identical white-balance and exposure settings. LFB staining was quantified as mean optical density using Fiji ImageJ v.1.52p (NIH, USA).

**Hematoxylin and Eosin Staining**

Hematoxylin and eosin (HE) staining was conducted according to standard histological procedures. Paraffin sections were deparaffinized in xylene and rehydrated through a graded ethanol series into distilled water. Sections were stained with hematoxylin for 5 min to visualize nuclei, then differentiated in 1 % acid ethanol for 3 s. After bluing and rinsing under running water, the cytoplasm was counterstained with eosin for 1 min. Finally, slides were dehydrated through ascending ethanol concentrations, cleared in xylene, and mounted with neutral balsam. All stained slides were air-dried overnight at room temperature before microscopic evaluation.

**Immunofluorescence Staining**

Immunofluorescence was performed on frozen sections. Brain tissues were fixed in 4% PFA solution for 48 h at 4 °C. After cryoprotection in graded sucrose (20 % and 30 % in 0.1 M PBS), tissues were embedded in optimal cutting temperature compound (Sakura Finetek, USA), rapidly frozen, and sectioned coronally at 20 μm using a cryostat microtome (Leica, Germany). Sections were permeabilized with 0.25 % Triton X-100 in PBS for 20 min and blocked with 3 % bovine serum albumin (BSA) for 1 h at room temperature. Primary antibodies diluted in blocking solution included rat anti-MBP (1:1000, ab7349, Abcam), rabbit anti-GST-pi (1:200, ab153949, Abcam), rabbit anti-NG2 (1:50, ab129051, Abcam), goat anti-CD31 (1:50, AF3628-SP, R&D Systems), and rabbit anti-p-CREB (1:500, 9198, CST). After PBS washes, sections were incubated with Alexa Fluor 488 or 594-conjugated secondary antibodies (Invitrogen) at 1:500 for 1 h at room temperature in the dark. Notably, endogenous rat IgG was detected directly using anti-rat Alexa Fluor 488-conjugated antibody, without the use of an additional primary antibody. Samples were mounted with anti-fade medium containing DAPI (P0131, Beyotime), and images were acquired using a fluorescence microscope (Keyence Corp., Osaka, Japan) and analyzed with Fiji ImageJ v.1.52p (NIH, USA).

**Immunohistochemical Staining**

Immunohistochemistry was performed on paraffin-embedded brain sections using an immunohistochemistry detection kit (KIT-9710, Maixin Biotechnology Co., Ltd., Fuzhou, China). After dewaxing and rehydration through graded alcohols, antigen retrieval was performed using EDTA buffer. Endogenous peroxidase activity was blocked using the peroxidase-blocking reagent supplied with the kit for 10 min at room temperature. After PBS washing, sections were incubated with the nonspecific staining blocking reagent supplied with the kit for 10 min at room temperature. Sections were subsequently incubated overnight at 4 °C with primary antibodies: mouse anti-GFAP (1:500, ab4648, Abcam), rabbit anti-Iba1 (1:500, 019-19741, Wako), and rabbit anti-p-PKA (1:100, 9621, CST). After washing, sections were incubated with the biotin-labeled goat anti-mouse/rabbit IgG polymer supplied with the detection kit for 20 min at room temperature, followed by incubation with streptavidin-peroxidase reagent for 10 min. After each incubation step, sections were washed three times with PBS. Immunoreactivity was visualized using a DAB substrate kit (Beijing Zhongshan Biotechnology Co., Ltd., China), and sections were dehydrated, cleared, and mounted with neutral gum for microscopic examination.

**Transmission Electron Microscopy**

For ultrastructural analysis, rats under deep anesthesia were transcardially perfused with PBS. After perfusion, brains were carefully harvested, and the corpus callosum was microdissected into 1 mm³ tissue blocks. The tissue blocks were immediately immersed in 2.5 % glutaraldehyde and fixed for 2 h at room temperature, followed by overnight fixation at 4 °C. After rinsing with buffer, the samples were post-fixed in 1% osmium tetroxide for 2 h at 4 °C. Samples were then stained en bloc with 1 % uranyl acetate for 1 h, dehydrated through graded ethanol, and embedded in epoxy resin. Ultrathin sections were double-stained with uranyl acetate and lead citrate to enhance contrast and examined using an HT-7700 transmission electron microscope (Hitachi, Japan).

**Western Blot**

Protein concentrations in corpus callosum samples were measured using a BCA protein assay kit (Beyotime Institute of Biotechnology, Shanghai, China). Equal amounts of protein were denatured, separated on 10 % SDS–polyacrylamide gels, and transferred to polyvinylidene fluoride membranes. Membranes were blocked for 1 h at room temperature with Fast Blocking Buffer (Bio-Rad Laboratories, Hercules, CA, USA) and incubated overnight at 4 °C with primary antibodies: rat anti-MBP (1:1000, ab7349, Abcam), mouse anti-Pde10a (1:1000, SC-515023, Santa), rabbit anti-p-PKA (1:1000, 9621, CST), rabbit anti-CREB (1:1000, 9197, CST), rabbit anti-p-CREB (1:500, 9198, CST), mouse anti-β-actin (1:5000, ab8226, Abcam), and mouse anti-GAPDH (1:10000, ab8245, Abcam). After washing, membranes were incubated for 1 h at room temperature with HRP-conjugated secondary antibodies (anti-rabbit, 1:10000, ab205718, Abcam; anti-mouse, 1:10000, ab205719, Abcam; anti-rat, 1:10000, SA00001-15, Proteintech). Protein bands were visualized with ECL substrate (PK10003, Proteintech) and imaged using a Bio-Rad Gel Imaging System (Version 3.73, Bio-Rad Laboratories, Hercules, CA, USA). Band intensities were quantified by densitometric analysis using Fiji ImageJ v.1.52p (NIH, USA).

**ELISA**

Blood samples were collected from the abdominal aorta into blood collection tubes and centrifuged to obtain serum. Serum concentrations of interleukin-1 beta (IL-1β) interleukin-6 (IL-6), and tumor necrosis factor-alpha (TNF-α) were determined using commercial ELISA kits. Cyclic adenosine monophosphate (cAMP) levels in the corpus callosum were also measured using a rat cAMP ELISA kit. All kits were obtained from Jianglaibio Co., Ltd. (Shanghai, China), and all assays were performed according to the manufacturer's instructions.

**Ultrasound Localization Microscopy**

Ultrasound localization microscopy (ULM) was performed to obtain super-resolved maps of the cerebral microvasculature. All imaging procedures were conducted in the medical ultrasound engineering laboratory at Tsinghua University. Rats were anesthetized with isoflurane and secured in a stereotaxic frame. A cranial window (2 mm × 6 mm) was surgically created between the bregma and lambda for ultrasound imaging. Functional ultrasound (FUS) imaging was conducted using an Iconeus One system (Iconeus One-256 channels, ICONEUS, France) equipped with a 15 MHz linear array transducer. The probe was positioned over the cranial window with ultrasound gel for acoustic coupling, and an ROI was selected under LiveView mode. For ULM, a bolus of a clinically approved microbubble contrast agent (TRANSCEND VIVOSCOPE Co., Ltd., Nanjing, China) was used. A fresh 100–200 μL bolus of diluted microbubble contrast agent was administered before acquisition of each coronal plane. Data were acquired using the SuperLoc 8V7PW sequence for 3 min per coronal brain plane, with 5 planes collected per rat to capture individual microbubble (MB) passages. After completion of ultrasound imaging, animals were euthanized under deep anesthesia and were not included in later longitudinal or terminal analyses.

Raw radiofrequency data were processed using the vendor's proprietary software to generate super-resolved vascular maps by localizing and tracking millions of MB trajectories. Maximum intensity projections of these trajectories were reconstructed to visualize the cerebrovasculature, and vascular density was quantified using Fiji ImageJ v.1.52p (NIH, USA) [S5].

**RNA-seq Transcriptomics Analysis**

Total RNA was extracted from rat corpus callosum tissue. mRNA was enriched using oligo(dT)-attached magnetic beads and subsequently fragmented. First-strand complementary DNA (cDNA) was synthesized using random hexamer primers, followed by second-strand synthesis. The resulting double-stranded cDNA was end-repaired, adenylated, and ligated to sequencing adapters. Polymerase chain reaction (PCR) amplification was then performed, and the libraries were quality-assessed and sequenced on the DNBSEQ platform with paired-end 150-bp reads. Raw sequencing reads were processed using SOAPnuke (v1.5.6) to remove low-quality bases, adapter sequences, and reads containing＞1% unknown nucleotides [S6]. Clean reads were aligned to the Rattus norvegicus reference genome (mRatBN7.2) using HISAT2 (v2.2.1). Gene expression levels were quantified with Bowtie2 (v2.3.4.3) and RSEM (v1.3.1). Differentially expressed genes (DEGs) were identified using DESeq2 (v1.4.5) [S7], with significance thresholds set at |log₂(fold change)|≥2 and an adjusted *P* value＜0.05.

**DIA-Based Proteomic Profiling and Analysis**

Total proteins were extracted from rat corpus callosum tissue using a lysis buffer containing 8 M urea and 1 % protease inhibitor cocktail, according to previously reported mass spectrometry-based proteomic workflows with minor modifications [S8, S10, S13]. Tissues were ground in liquid nitrogen, sonicated on ice, and centrifuged at 12,000 × g for 10 min at 4 °C. Protein concentration was determined using a BCA assay. Equal amounts of protein from each sample were adjusted to the same volume. One volume of pre-cooled acetone was first added and mixed by vortexing, followed by the addition of four volumes of pre-cooled acetone. Proteins were precipitated at −20 °C for 2 h and then centrifuged at 4,500 × g for 5 min. The resulting precipitates were washed two to three times with pre-cooled acetone and air-dried. Protein pellets were subsequently resuspended in 200 mM triethylammonium bicarbonate (TEAB) and dispersed by sonication. Trypsin was added at an enzyme-to-protein mass ratio of 1:50, and digestion was performed overnight. The samples were then reduced with 5 mM dithiothreitol (DTT) at 56 °C for 30 min and alkylated with 11 mM iodoacetamide (IAA) for 15 min at room temperature in the dark.

Tryptic peptides were dissolved in mobile phase A (0.1% formic acid in water) and separated using a Vanquish Neo ultra performance liquid chromatography system (Thermo Fisher Scientific) equipped with a reversed-phase analytical column, following established LC-MS/MS-based proteomic procedures [S9, S11, S12]. Mobile phase B consisted of 0.1% formic acid in 80% acetonitrile. Peptides were separated at a flow rate of 600 nL/min using the following gradient: 0–0.5 min, 4% B; 0.5–0.6 min, 4%–7.5% B; 0.6–8.1 min, 7.5%–30% B; 8.1–9.6 min, 30%–44% B; 9.6–10.0 min, 44%–55% B; 10.0–10.5 min, 55%–99% B; and 10.5–12.0 min, 99% B. The separated peptides were ionized using a capillary ion source and analyzed on a timsTOF HT mass spectrometer. The electrospray voltage was set to 1.6 kV. Both precursor and fragment ions were detected using a time-of-flight (TOF) analyzer. Data were acquired in data-independent acquisition parallel accumulation-serial fragmentation (dia-PASEF) mode. The full MS scan range was 300–1,500 m/z, followed by 12 PASEF MS/MS scans per acquisition cycle. The MS/MS scan range was 395–1,080 m/z, with an isolation window width of 15 m/z.

Raw data were processed using DIA-NN (v1.8) against the Rattus norvegicus UniProt database (47,921 entries). Search parameters included trypsin/P digestion with up to one missed cleavage, fixed modifications of N-terminal methionine excision and carbamidomethylation on cysteine, and a 1 % false discovery rate at the precursor level. Proteins with a fold change (FC)＞1.5 and a *P* value＜0.05 were considered significantly upregulated, whereas those with a FC＜1/1.5 and a *P* value < 0.05 were considered significantly downregulated.

**Integrative Analysis**

Given the stringent multiple-testing correction applied to transcriptomic data, which may exclude certain biologically relevant changes, a direction-based filtering strategy was adopted for the integrative analysis. Specifically, transcripts with a threshold of log_2_FC＞0 or log_2_FC＜0 and an adjusted *P* value＜0.05 were retained to preserve transcriptional signals associated with protein expression changes, aiming to identify molecules exhibiting concordant expression patterns across the two omics. Functional enrichment analysis was subsequently performed according to distinct transcript and protein regulatory relationships. Key molecules were further validated using RT-qPCR and western blot analysis.

**References for Supplementary Methods**

S1.	Lin CH, Wu YT, Wei JY, Shih YC, Chao YP, Lai YJ, Chiu YL, Chuang YF. Frailty subtypes and white matter alterations in older adults without dementia. *Psychiatry and Clinical Neurosciences* 2025;79:677–684.

S2.	Soares JM, Marques P, Alves V, Sousa N. A hitchhiker’s guide to diffusion tensor imaging. *Frontiers in Neuroscience* 2013;7:31.

S3.	Winston GP. The physical and biological basis of quantitative parameters derived from diffusion MRI. *Quantitative Imaging in Medicine and Surgery* 2012;2:254–265.

S4.	Marano G, Kotzalidis GD, Anesini MB, Barbonetti S, Rossi S, Milintenda M, Restaino A, Acanfora M, Traversi G, Veneziani G*, et al.* Exploring the autistic brain: a systematic review of diffusion tensor imaging studies on neural connectivity in autism spectrum disorder. *Brain Sciences* 2025;15:824.

S5. Nyúl-Tóth Á, Negri S, Sanford M, Jiang R, Patai R, Budda M, Petersen B, Pinckard J, Chandragiri SS, Shi H, et al. Novel intravital approaches to quantify deep vascular structure and perfusion in the aging mouse brain using ultrasound localization microscopy (ULM). *Journal of Cerebral Blood Flow and Metabolism* 2024;44:1378–1396.

S6. Chen Y, Chen Y, Shi C, Huang Z, Zhang Y, Li S, Li Y, Ye J, Yu C, Li Z, et al. SOAPnuke: a MapReduce acceleration-supported software for integrated quality control and preprocessing of high-throughput sequencing data. *Gigascience* 2018;7:1–6.

S7. Love MI, Huber W, Anders S. Moderated estimation of fold change and dispersion for RNA-seq data with DESeq2. *Genome Biology* 2014;15:550.

S8. Chen H, Li Y, Li H, Chen X, Fu H, Mao D, Chen W, Lan L, Wang C, Hu K, et al. NBS1 lactylation is required for efficient DNA repair and chemotherapy resistance. *Nature* 2024;631:663–669.

S9. Li H, Liu C, Li R, Zhou L, Ran Y, Yang Q, Huang H, Lu H, Song H, Yang B, et al. AARS1 and AARS2 sense L-lactate to regulate cGAS as global lysine lactyltransferases. *Nature* 2024;634:1229–1237.

S10. Chen Y, Wu J, Zhai L, Zhang T, Yin H, Gao H, Zhao F, Wang Z, Yang X, Jin M, et al. Metabolic regulation of homologous recombination repair by MRE11 lactylation. *Cell* 2024;187:294–311.e21.

S11. Zong Z, Xie F, Wang S, Wu X, Zhang Z, Yang B, Zhou F. Alanyl-tRNA synthetase, AARS1, is a lactate sensor and lactyltransferase that lactylates p53 and contributes to tumorigenesis. *Cell* 2024;187:2375–2392.e33.

S12. Liang L, Kuang X, He Y, Zhu L, Lau P, Li X, Luo D, Gong L, Zhou W, Zhang F, et al. Alterations in PD-L1 succinylation shape anti-tumor immune responses in melanoma. *Nature Genetics* 2025;57:680–693.

S13. Lao Y, Cui X, Xu Z, Yan H, Zhang Z, Zhang Z, Geng L, Li B, Lu Y, Guan Q, et al. Glutaryl-CoA dehydrogenase suppresses tumor progression and shapes an anti-tumor microenvironment in hepatocellular carcinoma. *Journal of Hepatology* 2024;81:847–861.

**Supplementary Table**

**Table S1.** Primer sequence used for RT-qPCR.

| **Gene** | **Forward Primer** | **Reverse Primer** |
| --- | --- | --- |
| R-Pde10a | AATCTAGTGAACGCCGACCG | GACTTCTCCCGTTCTTGCCA |
| R-PKA | GCAGGAGAGCGTGAAAGA | TCCAACTGGGCTGTATTCTG |
| R-CREB | CAAGTTGTTGTTCAAGCTGCCT | TAGACGAACCTCTCTCTTTCGTG |
| R-GAPDH | CCGCATCTTCTTGTGCAGTG | ATCCGTTCACACCGACCTTC |
| R-ACTB | CTGTGTGGATTGGTGGCTCT | AGCTCAGTAACAGTCCGCCT |

Abbreviations: R denotes *Rattus norvegicus*.

**Supplementary Figures**

**FIGURE S1** Transcriptomic profiling reveals cAMP signaling enrichment in white matter lesions of CSVD. (A) Principal component analysis (PCA) of transcriptomic data demonstrates clear segregation between SHRs and WKY groups. (B) Volcano plot depicting differentially expressed genes (DEGs) between SHRs and WKY groups; red indicates upregulated genes, blue indicates downregulated genes, and gray indicates non-significant genes. (C–E) Gene Ontology (GO) enrichment analysis of DEGs showing significantly enriched (C) biological processes, (D) cellular components, and (E) molecular functions. (F) Kyoto Encyclopedia of Genes and Genomes (KEGG) pathway enrichment highlights pathways related to neurodegeneration and vascular dysfunction. (G) Gene set enrichment analysis (GSEA) confirms significant enrichment of the cAMP signaling pathway in SHRs. The green line represents the enrichment score (ES), with the Y-axis peak indicating the maximum ES value. n = 6 per group.

**FIGURE S2** Quality control and proteomic profiling of corpus callosum in SHR and WKY groups. (A) Total identified peptides, unique peptides, identified proteins, and comparable proteins in the proteomic analysis. (B) Quantifiable proteins and their cumulative distribution across WKY and SHR samples. (C) Log10 intensity distribution for the proteome across samples, with intensity categories highlighted by different colors, reflecting the protein abundance in each sample. n = 6 per group.

**FIGURE S3** Proteomic profiling highlights Pde10a as an upregulated candidate associated with cAMP signaling in CSVD-related WMLs. (A) PCA of proteomic data demonstrates clear segregation between SHR and WKY groups. (B) Bar plot summarizing differentially expressed proteins (DEPs): 57 upregulated (red) and 38 downregulated (blue). (C) Volcano plot showing DEPs between SHRs and WKY groups; red indicates significantly upregulated proteins, blue indicates downregulated proteins, and gray indicates non-significant proteins. (D) Heatmap of all identified DEPs illustrating intergroup clustering. (E) GO enrichment of DEPs in biological process categories. (F) KEGG pathway enrichment highlighting metabolic, immune, and neural signaling pathways. (G) Chord diagram illustrating the relationships between DEPs and enriched KEGG pathways. (H) Protein–protein interaction (PPI) network of DEPs; red nodes represent upregulated proteins, blue nodes represent downregulated proteins, and node size correlates with interaction degree. (I) Stratification of DEPs into four fold-change intervals (Q1–Q4) based on fold-change magnitude, from lowest (Q1) to highest (Q4). (J) Biological processes of GO enrichment terms associated with DEPs across intervals. (K) KEGG enrichment for Q4 proteins showing marked enrichment of the cAMP signaling pathway. n = 6 per group.
